# Primary Pericardial Synovial Sarcoma: An Extremely Rare Cardiac Neoplasm

**DOI:** 10.7759/cureus.14583

**Published:** 2021-04-20

**Authors:** Ammar Farook Chapra, Abdul Majeed Maliyakkal, Vamanjore A Naushad, Hanee S Valiyakath, Mustafa S Ahmed

**Affiliations:** 1 Internal Medicine, Hamad Medical Corporation, Doha, QAT; 2 General Internal Medicine, Hamad Medical Corporation, Doha, QAT; 3 Clinical Medicine, Weill Cornell, Doha, QAT; 4 Clinical Medicine, College of Medicine Qatar University, Doha, QAT; 5 Radiology, Hamad Medical Corporation, Doha, QAT; 6 Medicine, Hamad Medical Corporation, Doha, QAT

**Keywords:** pericardial effusion, pericardial synovial sarcoma, cardiac tumor

## Abstract

Primary pericardial tumors are an entity that is infrequently encountered and may be a cause of pericardial effusion. Primary synovial sarcomas of the pericardium are even rarer malignant invasive tumors that are a challenge to recognize due to their vague presentation and difficulty in diagnosing non-invasively. Here, we report a case of a 48-year-old gentleman of South Asian descent, who was incidentally found to have pericardial and bilateral pleural effusions and subsequently diagnosed to have primary pericardial synovial sarcoma.

## Introduction

Primary cardiac tumors are an extremely rare entity with a prevalence rate of 0.02% to 0.056% [1-2]. Primary tumors of the pericardium are even rarer, and their prevalence ranges from 0.001% to 0.007% [3]. Primary pericardial tumors may be benign or malignant. Benign tumors include pericardial cysts, lipomas, lipoblastomas, paragangliomas, germ-cell tumors, hemangiomas, fibromas, and inflammatory pseudo-tumors. Pericardial cysts are the most common benign tumors followed by lipomas. Primary malignant pericardial neoplasms include mesotheliomas, a wide variety of sarcomas, lymphomas, and primitive neuroectodermal tumors amongst which mesotheliomas are the most common [4]. In this article, we present a rare case of primary pericardial synovial sarcoma with a review of the literature.

## Case presentation

A 48-year-old gentleman of South Asian descent who had no prior medical illness was referred to our hospital from a peripheral clinic for the evaluation of incidental sonographic finding of pericardial and bilateral pleural effusions while undergoing evaluation for renal colic. On admission, he was primarily complaining of right flank pain associated with dysuria of two days duration. On a detailed review of history, he admitted having shortness of breath associated with a mild nonproductive cough of 10 days duration. Also, he was complaining of anorexia and paroxysmal nocturnal dyspnea (PND). On examination his vital signs on admission were: pulse rate 103/minute, blood pressure 122/80 mmHg, respiratory rate 18/minute, temperature 37°C, and oxygen saturation 99% breathing on room air. Jugular venous pulse was not raised. Systemic examination revealed a comfortably lying patient without any distress. Respiratory system examination showed dullness on percussion with decreased intensity of breath sounds over the left infra-scapular area. Other system examinations, including the cardiovascular system, were unremarkable.

His initial laboratory investigations were unremarkable except for a mildly elevated C-reactive protein (CRP) of 30 mg/L (Table 1).

**Table 1:** Showing baseline laboratory results WBC-White blood cell count, RBC-Red blood cell count, ALT-Alanine aminotransferase, AST-Aspartate aminotransferase, LDH-Lactate dehydrogenase

Test	Result	Normal Range
WBC	10.6 x10^3/uL	4.0-10.0
RBC	5.7 x10^6/uL	4.5-5.5
Hemoglobin	13.1 gm/dL	13.0-17.0
Hematocrit	40.2 %	40.0-50.0
Platelet	302 x10^3/uL	150-400
Absolute Neutrophil count (ANC)	9.4 x10^3/uL	2.0-7.0
Lymphocytes count	0.66 x10^3/uL	1.00-3.00
Urea	7.6 mmol/L	3.2-7.4
Creatinine	86 umol/L	64-110
Total Protein	64 gm/L	64-83
Albumin	40 gm/L	35-50
Alkaline Phosphatase	64 U/L	40-150
ALT	42 U/L	0-55
AST	34 U/L	5-34
LDH	259 U/L	125-220
High Sensitivity Troponin-T	6.05 ng/L	0.00-14.00
Glucose	5.0 mmol/L	3.3-5.5
C-Reactive Protein	30 mg/L	0-5
Procalcitonin	0.14 ng/mL	

**Figure 1:**
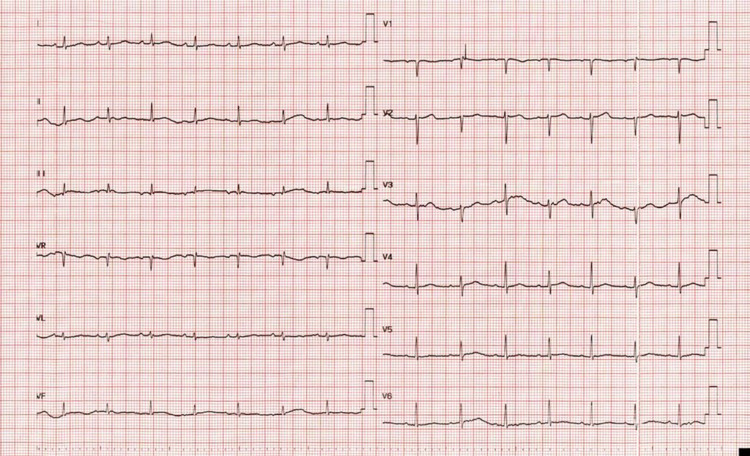
Electrocardiogram showing electrical alternans

Electrocardiogram (ECG) on admission showed sinus tachycardia with electrical alternans (Figure 1).

**Figure 2:**
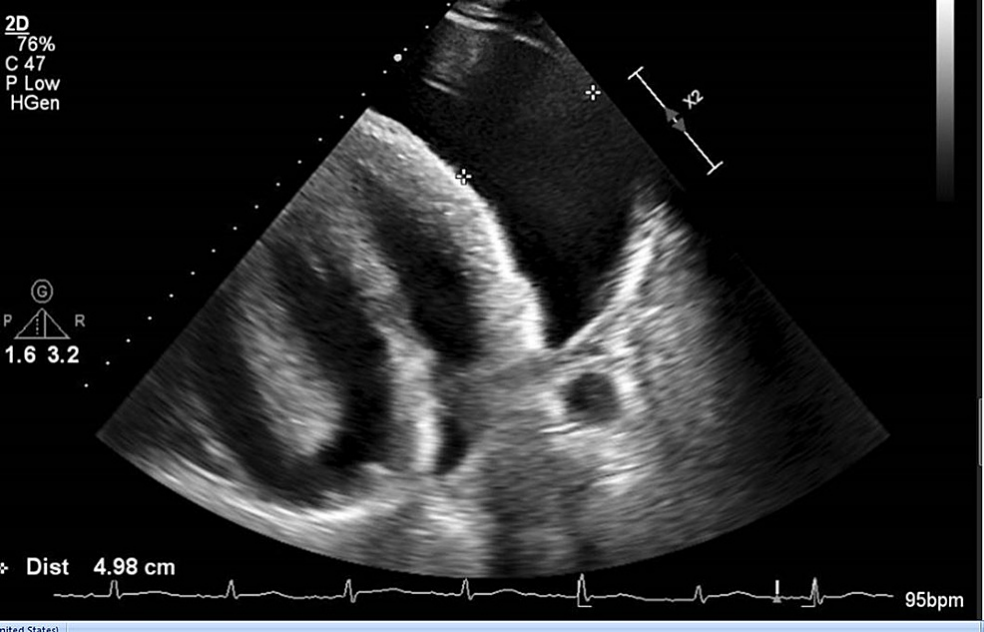
Transthoracic echocardiogram showing large pericardial effusion

Chest X-ray demonstrated an enlarged cardiac silhouette with blunting of the left costophrenic angle. Transthoracic echocardiography revealed a large pericardial effusion suggestive of cardiac tamponade (Figure 2).

Abdominal sonography showed a right renal calculus measuring 7.6 mm without hydronephrosis. The patient underwent emergent pericardiocentesis with drainage of 950 ml of sanguineous fluid and a left-sided pericardial drain was subsequently inserted. His shortness of breath improved following pericardiocentesis and flank pain resolved with conservative management. Pericardial fluid analysis revealed an exudative effusion with no bacteria or neoplastic cells (Table 2).

**Table 2:** Results of pericardial fluid analysis WBC-White blood cell count, RBC-Red blood cell, LDH- Lactate dehydrogenase

Pericardial fluid analysis	Result
Color	Red
Appearance	Bloody
WBC	6,125 /uL
RBC	646,125 /uL
Lymphocytes	57.0 %
Monocytes	29.0 %
Macrophages	8.0 %
Mesothelial cells	2.0 %
Glucose	6.9 mmol/L
LDH	273.0 U/L
pH	7.471
Protein	29.0 gm/L
Albumin	18.0 gm/L
Cytology	Lymphohistiocytic effusion. No malignant cells seen
Fluid Culture	No growth

Initially, a possibility of tuberculous etiology was considered; however, workup was negative for the presence of acid-fast bacilli (AFB) in the smear, tuberculosis polymerase chain reaction (TB-PCR), and culture. Quantiferon-TB Gold (QFT) was also negative. Screening for common autoimmune causes of serositis did not yield any positive results.

His COVID-19 PCR was also negative. Meanwhile, the patient was given symptomatic management with ibuprofen 600 mg every six hours and colchicine 0.5 mg daily.

**Figure 3:**
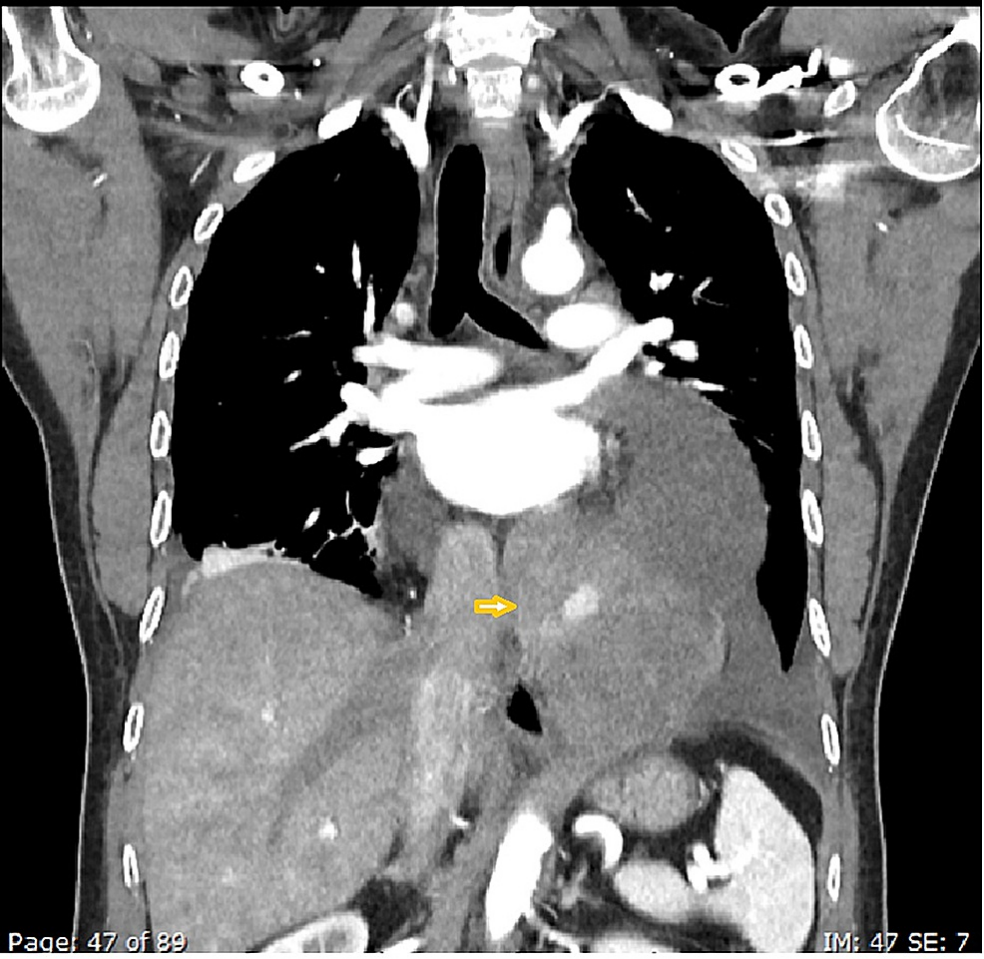
Coronal contrast CT (arterial phase) images showing a large, heterogeneously enhancing pericardial mass (arrowhead)

**Figure 4:**
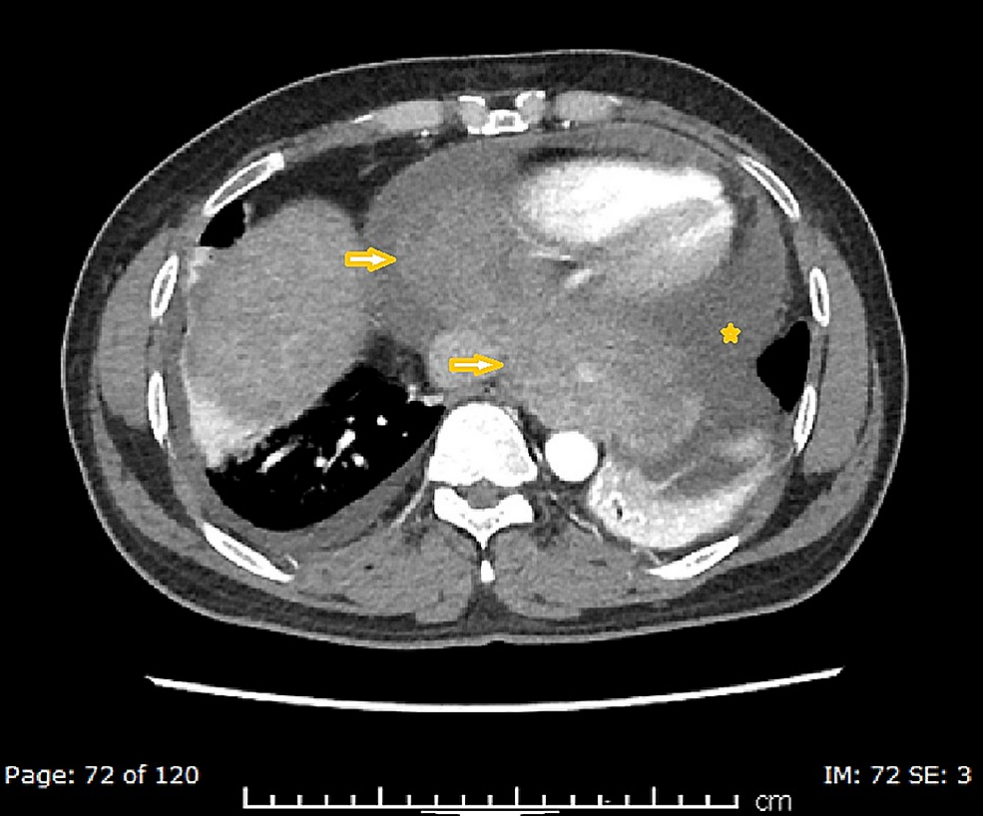
Axial contrast CT (arterial phase) images showing a large, heterogeneously enhancing pericardial mass (arrowheads) with pericardial effusion (star)

**Figure 5:**
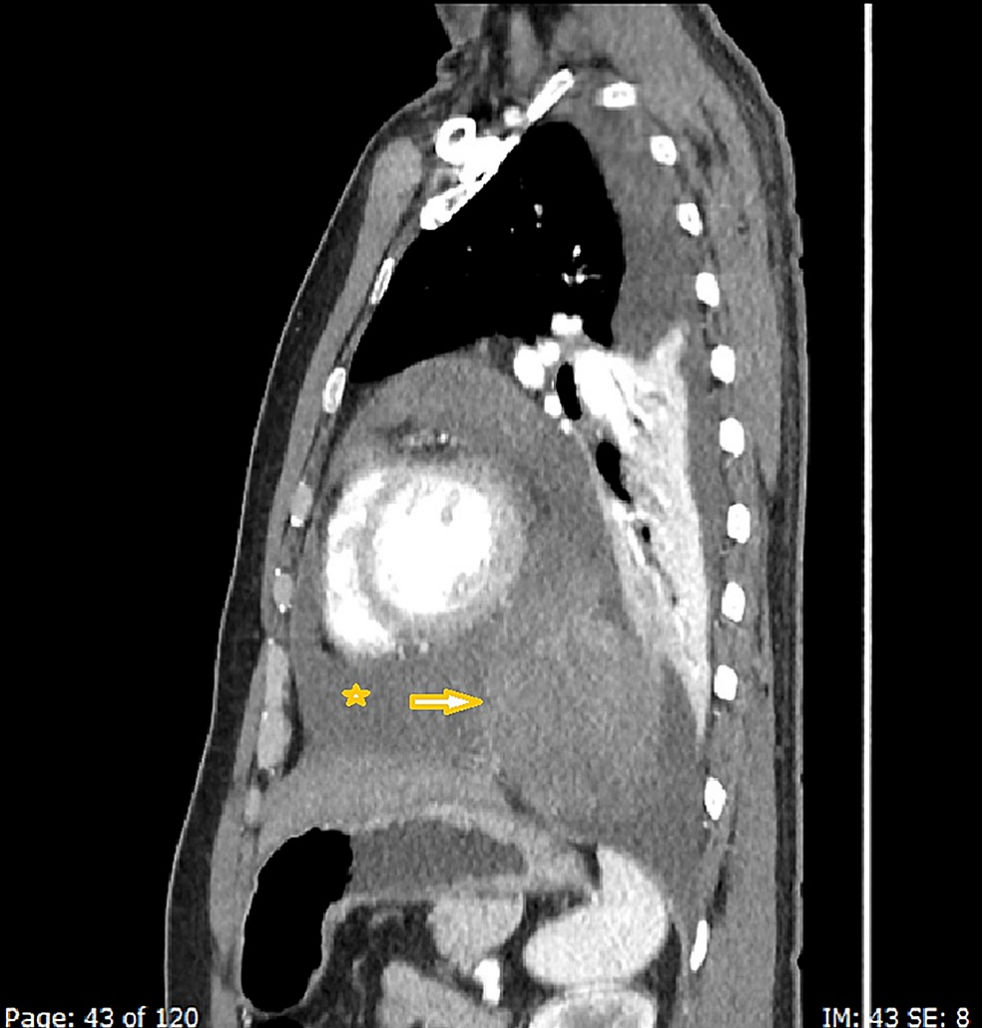
Sagittal contrast CT (arterial phase) images showing a large, heterogeneously enhancing pericardial mass (arrowhead) with pericardial effusion (star)

With the initial workup being unable to delineate the cause of serositis, a computed tomography (CT) scan of the chest, abdomen, and pelvis was performed, which showed a well-defined heterogeneous structure measuring 13.5 x 5 cm on the lower aspect of the pericardium extending into the left side of the thorax, abutting the cardiac border and the anterior part of the aorta without any infiltration (Figures 3-5).

The radiological findings and the sanguineous nature of the pericardial fluid indicated an organized hematoma versus a neoplastic etiology. The CT also confirmed bilateral pleural effusion, more on the left side. No free fluid was seen in the abdomen. Subsequent diagnostic pleurocentesis from the left pleural effusion yielded 80 ml of sanguineous fluid, which was exudative as well. It was predominantly lymphohistiocytic, without any growth of bacteria, AFB, or neoplastic cells.

**Figure 6:**
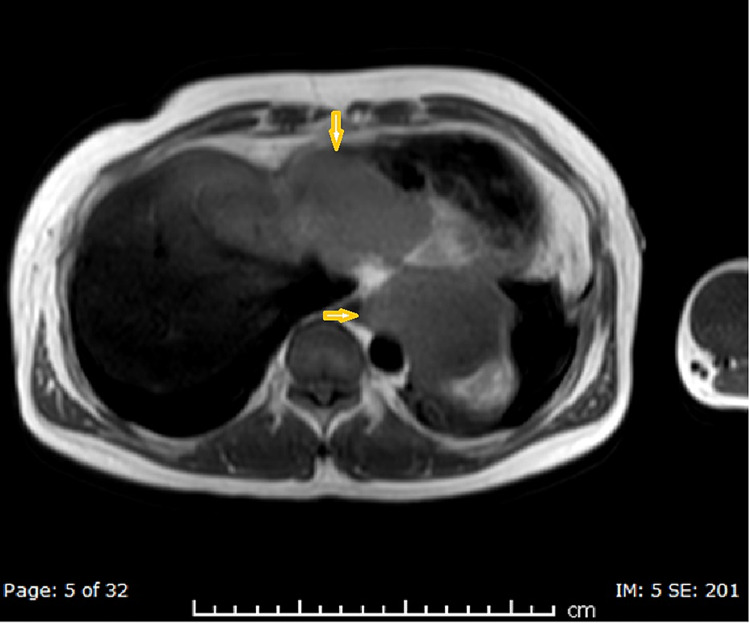
MRI T2-weighted axial image showing a pericardial mass lesion (arrowheads)

**Figure 7:**
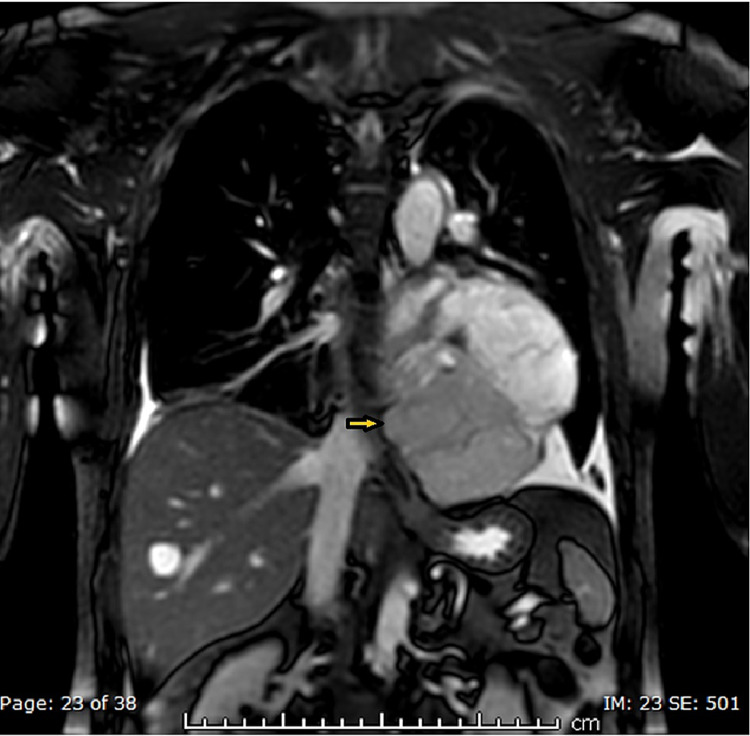
MRI-STIR sequence coronal image showing a pericardial mass lesion (arrowhead) STIR: short-TI inversion recovery

**Figure 8:**
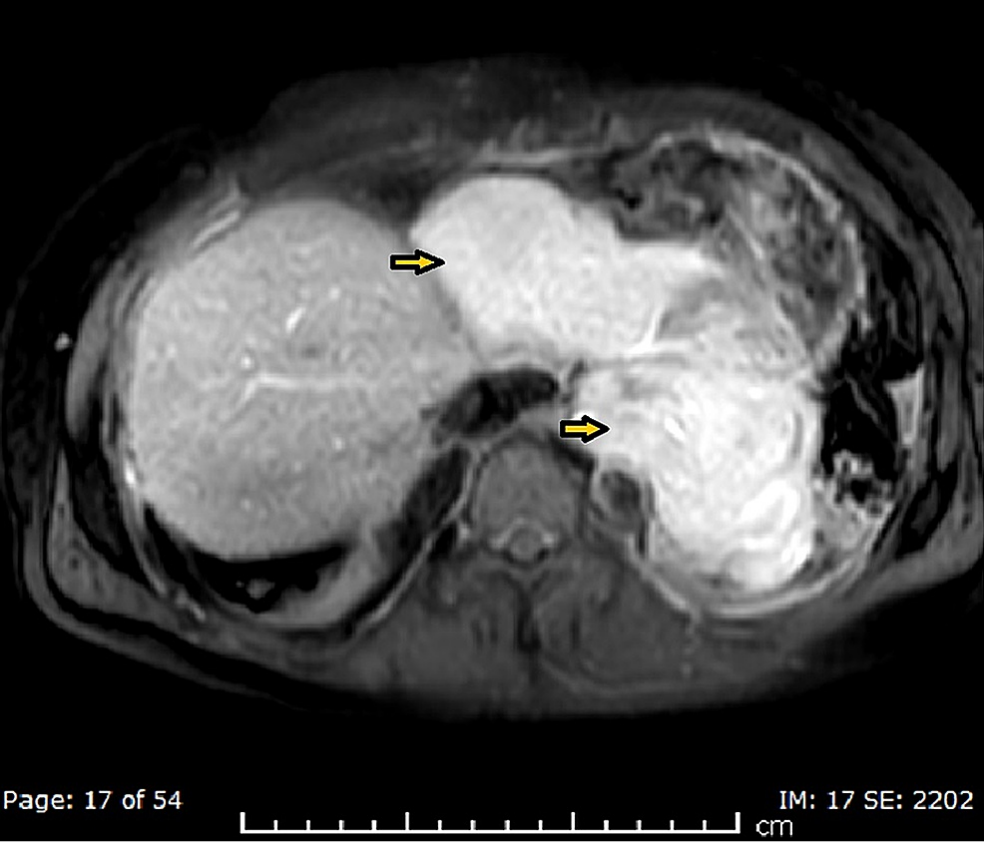
MRI post-contrast axial image showing a pericardial mass lesion (arrowheads)

The patient underwent further characterization of the suspected hematoma/neoplasm with a cardiac magnetic resonance imaging (MRI), which showed three well-defined pericardial soft tissue masses exhibiting features of local invasiveness on the left inferior pleura and left hemidiaphragm without infiltration, highly suggestive of a neoplastic origin; however, atypical hemangioma was still a consideration (Figures 6-8).

**Figure 9:**
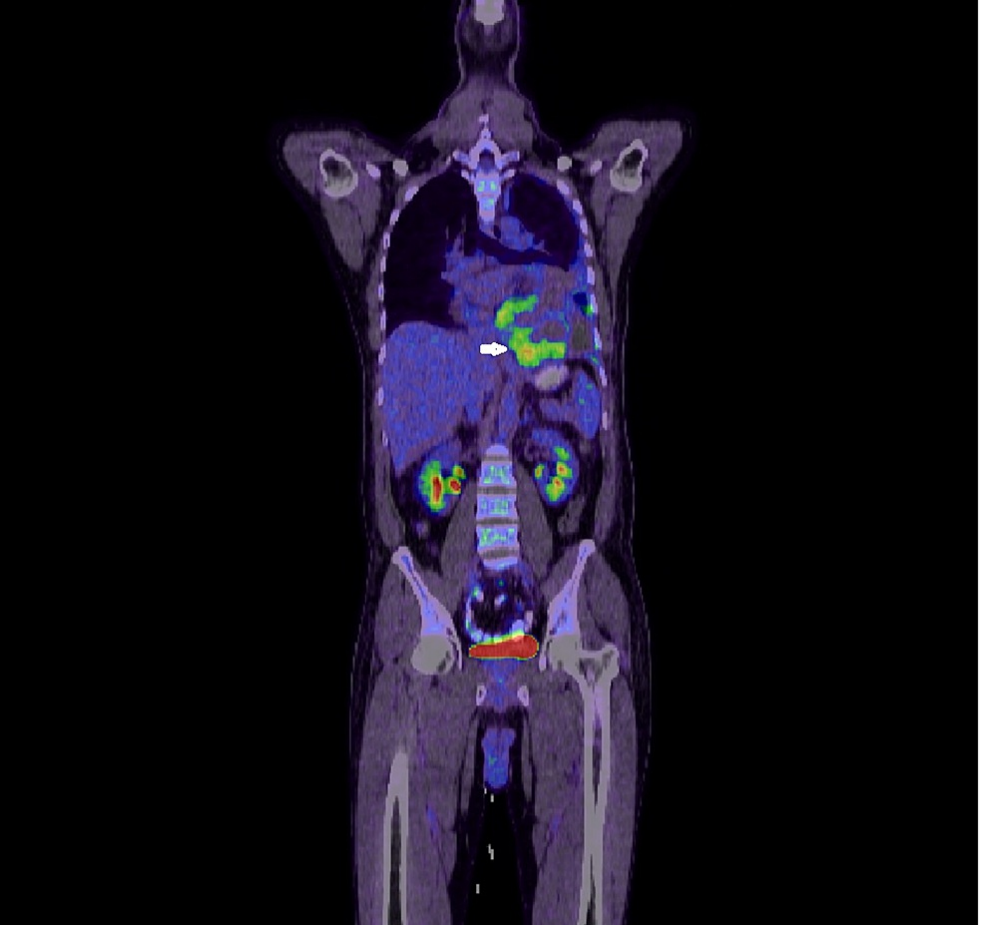
PET scan coronal section image showing increased uptake by a pericardial mass lesion (arrowhead) PET: positron emission tomography

**Figure 10:**
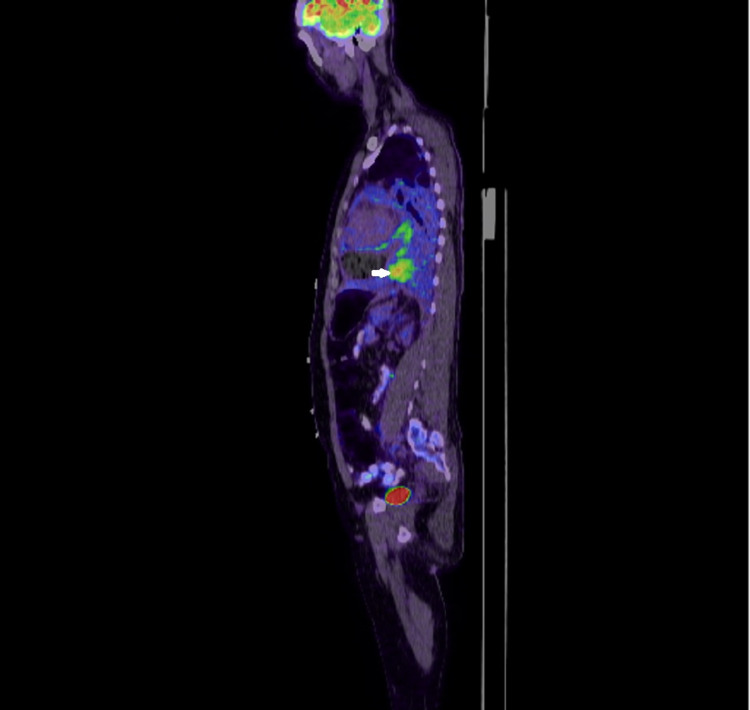
PET scan sagittal section image showing increased uptake by a pericardial mass lesion (arrowhead) PET: positron emission tomography

**Figure 11:**
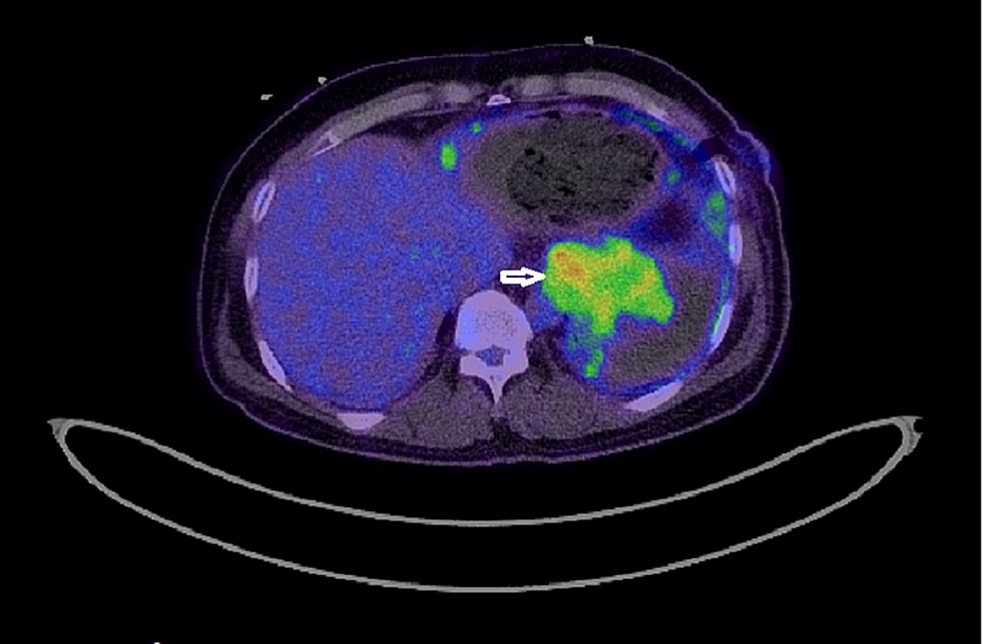
PET scan axial section image showing increased uptake by a pericardial mass lesion (arrowhead) PET: positron emission tomography

Follow-up echocardiography after three days revealed the recurrence of a large circumferential pericardial effusion without features of tamponade. The patient remained clinically well without any new complaints; however, his hemoglobin was progressively declining from 13.1 gm/dL to 7.5 gm /dL, which was managed with packed red blood cells (PRBC) transfusion. A positron emission tomography (PET) scan confirmed increased fluorodeoxyglucose (FDG) uptake in the pericardial and pleural masses without any uptake elsewhere, indicating a primary pericardial-pleural pathology (Figures 9-11).

The patient subsequently underwent a left video-assisted thoracoscopic (VATS) biopsy of the pericardial mass. Histopathological examination of the tissue samples from the pericardial and pleural segments of the mass showed fascicles of spindle cells with moderate cellular atypia, frequent mitoses, and necrosis most compatible with a diagnosis of synovial sarcoma. By immunohistochemistry, the cells were strongly positive for cytokeratin AE1/AE3, FLI-1, cytokeratin 8/18, TLE-1, BCL-2, CD99, cytokeratin 7, and D2-40 and showed patchy positivity with epithelial membrane antigen, WT-1, cytokeratin 5/6, Gata-3, and ERG. The proliferative index (immunostaining with Ki67) was estimated to be around 60%. Fluorescence in-situ hybridization (FISH) testing was positive for synovial sarcoma translocation on chromosome 18 (SS18) rearrangement and negative for Ewing sarcoma breakpoint region 1 (EWSR1) rearrangement, further confirming the diagnosis of a locally advanced primary synovial sarcoma of the pericardium.

## Discussion

Primary pericardial sarcomas are very uncommon and aggressive tumors. They form approximately 13% of all primary cardiac sarcomas [5]. They have been classified into subtypes based on histopathological appearance, which includes angiosarcoma, liposarcoma, rhabdomyosarcoma, synovial sarcoma, and undifferentiated sarcoma [6]. Angiosarcoma is the most common type followed by undifferentiated sarcoma and leiomyosarcoma.

Like other primary pericardial neoplasms, the primary pericardial synovial sarcomas will also have varied clinical presentations. The symptoms include dyspnea, chest pain, cough, palpitation, and fever. The symptoms are mainly due to the presence of pericarditis, pericardial effusion, and cardiac tamponade.

Evaluation of pericardial synovial sarcomas includes chest radiography, which might reveal cardiomegaly or the presence of a widened mediastinum [7]. Echocardiographic findings include pericardial effusion or a thickened pericardium [8]. CT scan will detect the location of the tumor and will show the local invasion or distant metastasis. Magnetic resonance imaging is considered a better imaging tool than a CT scan, as it provides higher resolution and can detect invasion of the myocardium [7-9].

Very few cases of synovial sarcoma have been reported in the past. A literature review by Cheng et al. on 14 published cases of primary pericardial synovial sarcoma showed that the median age at the time of diagnosis was 32 years, with a preponderance in males [7], and the mean size of the tumor was 10.6 cm [10]. The most common symptom was dyspnea followed by chest pain and cough, with most of the symptoms attributed to cardiac tamponade. Our patient was in his late forties and his renal colic may be coincidental, the severity of which might have precluded/distracted the patient from complaining of chest symptoms initially.

## Conclusions

Although primary pericardial synovial sarcoma is extremely rare, it should be considered as one of the differential diagnoses in the evaluation of patients presenting with pericardial effusion of an unknown etiology. CT scan can detect the location of the tumor as well as metastasis and local invasion; however, for detecting myocardial invasion, MRI is considered to be a better imaging tool.

## References

[1] Lam KY, Dickens P, Chan AC (1993). Tumors of the heart. A 20-year experience with a review of 12,485 consecutive autopsies. Arch Pathol Lab Med.

[2] Reynen K (1996). Frequency of primary tumors of the heart. Am J Cardiol.

[3] Restrepo CS, Vargas D, Ocazionez D, Martínez-Jiménez S, Betancourt Cuellar SL, Gutierrez FR (2013). Primary pericardial tumors. Radiographics.

[4] Patel J, Sheppard MN (2010). Pathological study of primary cardiac and pericardial tumours in a specialist UK Centre: surgical and autopsy series. Cardiovasc Pathol.

[5] Burke AP, Cowan D, Virmani R (1992). Primary sarcomas of the heart. Cancer.

[6] Dillman JR, Pernicano PG, McHugh JB (2010). Cross-sectional imaging of primary thoracic sarcomas with histopathologic correlation: a review for the radiologist. Curr Probl Diagn Radiol.

[7] Grebenc ML, Rosado de Christenson ML, Burke AP, Green CE, Galvin JR (2000). Primary cardiac and pericardial neoplasms: radiologic-pathologic correlation. Radiographics.

[8] Lamba G, Frishman WH (2012). Cardiac and pericardial tumors. Cardiol Rev.

[9] Randhawa K, Ganeshan A, Hoey ET (2011). Magnetic resonance imaging of cardiac tumors: part 2, malignant tumors and tumor-like conditions. Curr Probl Diagn Radiol.

[10] Cheng Y, Sheng W, Zhou X, Wang J (2012). Pericardial synovial sarcoma, a potential for misdiagnosis: clinicopathologic and molecular cytogenetic analysis of three cases with literature review. Am J Clin Pathol.

